# Real-time imaging of intestinal bacterial β-glucuronidase activity by hydrolysis of a fluorescent probe

**DOI:** 10.1038/s41598-017-03252-4

**Published:** 2017-06-09

**Authors:** Michael Chen, Kai-Wen Cheng, Yi-Jou Chen, Chang-Hung Wang, Ta-Chun Cheng, Kuo-Chien Chang, An-Pei Kao, Kuo-Hsiang Chuang

**Affiliations:** 1 Ph.D. Program for the Clinical Drug Discovery from Botanical Herbs, Taipei Medical University, Taipei, Taiwan; 2 Institute of Biomedical Sciences, National Sun Yat-Sen University, Kaohsiung, Taiwan; 3 Center for Biomarkers and Biotech Drugs, Kaohsiung Medical University, Kaohsiung, Taiwan; 4 Department of Orthopedics, Tri-service General Hospital, Taipei, Taiwan; 5 Stemforce Biotechnology Co., Ltd, Chiayi City, Taiwan; 6 Graduate Institute of Pharmacognosy, Taipei Medical University, Taipei, Taiwan; 7 Ph.D Program in Biotechnology Research and Development, Taipei Medical University, Taipei, Taiwan; 8 The Ph.D. Program of Translational Medicine, Taipei Medical University, Taipei, Taiwan

## Abstract

Intestinal bacterial β-glucuronidase (βG) hydrolyzes glucuronidated metabolites to their toxic form in intestines, resulting in intestinal damage. The development of a method to inhibit βG is thus important but has been limited by the difficulty of directly assessing enzyme activity in live animals. Here, we utilized a fluorescent probe, fluorescein di-β-D-glucuronide (FDGlcU), to non-invasively image the intestinal bacterial βG activity in nude mice. *In vitro* cell-based assays showed that the detection limit is 10^4^ colony-forming units/well of βG-expressing bacteria, and that 7.81 ng/mL of FDGlcU is enough to generate significant fluorescent signal. In whole-body optical images of nude mice, the maximum fluorescence signal for βG activity in intestines was detected 3 hours after gavage with FDGlcU. Following pretreatment with a bacterial βG inhibitor, the fluorescence signal was significantly reduced in abdomens and excised intestines images. For a 4-day antibiotic treatment to deplete intestinal bacteria, the FDGlcU-based images showed that the βG activity was decreased by 8.5-fold on day 4 and then gradually increased after treatment stopped. The results suggested that FDGlcU-based imaging revealed the *in vitro* and *in vivo* activity of intestinal bacterial βG, which would facilitate pharmacodynamic studies of specific bacterial βG inhibitors in animal studies.

## Introduction

Bacterial β-glucuronidase (βG), a glycosidase widely present in intestinal microflora, has been reported to influence metabolism and detoxification in mammals. During detoxification in the liver, xenobiotic and harmful substances are conjugated with glucuronides by uridine 5′-diphospho-glucuronosyltransferase to become more hydrophilic metabolites which can be secreted into the intestine in bile and then eliminated from the body^1^. However, the glucuronides are catalytically hydrolyzed by intestinal bacterial βG. As a result, the metabolites are converted back into the hydrophobic and toxic form, causing intestinal damage^2^. Also, the involvement of intestinal bacterial βG in drug metabolism causes drug-induced intestinal injury. For example, in colorectal cancer patients treated with CPT-11, bacterial βG converts SN38-glucuronide (SN-38G, a nontoxic metabolite of CPT-11) to its toxic form SN-38, resulting in intestinal mucosal damage and diarrhea^3, 4^. In one previous study, one-third of patients who received CPT-11 experienced severe diarrhea, resulting in a dose reduction or the discontinuation of the CPT-11 treatment^5^. Other studies involving non-steroidal anti-inflammatory drugs have also reported that those metabolites are reactivated by bacterial βG and cause enteropathy^6–8^. Additionally, excessive catalytic activity on the part of intestinal bacterial βG has also been suggested to act as a contributor to colorectal carcinogenesis^9^. Hence, prophylaxis against bacterial βG-mediated intestinal damage is urgently needed in order to relieve the side effects of treatment and the consequent discomfort of patients.

To date, various strategies to reduce the enzyme activity of intestinal bacterial βG have been developed to prevent the related intestinal damage. The elimination of intestinal flora by using antibiotics has been shown to be effective in animal experiments and clinical trials^4, 10^. Another strategy consists of directly blocking βG through the use of specific inhibitors. Saccharic acid 1.4-lactone^11^ and a bacterial βG-specific inhibitor^12^, for instance, are capable of reducing the incidence rate of CPT-11-induced diarrhea in animal models. When developing a preventive therapy for intestinal bacterial βG, however, it is difficult to directly evaluate the attenuation of the enzyme activity and design a dose regimen. For the detection of intestinal bacterial βG in animal models, commercial substrate-based reagents and kits that can provide *ex vivo* measurements of βG activity in feces are available^13, 14^. More specifically, the hydrolysis of 4-nitrophenyl β-D-glucuronide (PNPG) or phenolphthalein β-D-glucuronide (PHTG) can be measured with spectrophotometric assays, while the hydrolysis of 4-methylumbelliferyl β-D-glucuronide (4-MUG) can be measured with fluorometric assays. Nevertheless, the βG in feces is not truly representative of the enzyme activity in the entire intestine of a live animal model. However, a method by which to precisely detect intestinal bacterial βG activity in animal models is still unavailable.

In this study, we utilized fluorescein Di-β-D-Glucuronide (FDGlcU) to non-invasively detect bacterial βG activity in the murine intestine via optical imaging. FDGlcU is non-fluorescent when the fluorescein is conjugated with two mono-glucuronides, but it shows a high level of fluorescent activity (λ_ex_ = 480 nm/λ_em_ = 514 nm) after βG removes the mono-glucuronides. It has thus been used to provide *in vitro* measurements of the catalytic activity of βG. We hypothesized, therefore, that the catalytic hydrolysis of FDGlcU could indicate the *in vivo* activity of βG in the murine intestine, and in the present study, we tested the correlation between βG activity and the number of bacteria *in vitro*. During *in vivo* imaging experiments, nude mice were oral gavaged with FDGlcU prior to a serial optical imaging. Then, the effect of a specific bacterial βG inhibitor^12^, 1-((6,8-Dimethyl-2-oxo-1,2-dihydroquinolin-3-yl)methyl)-3-(4-ethoxyphenyl)-1-(2-hydroxyethyl)thiourea (termed eβG inhibitor), was evaluated with FDGlcU-based imaging after oral administration of the inhibitor. Finally, we used FDGlcU-based imaging to evaluate the reduction of βG-expressing bacteria by an antibiotic treatment, and the results suggested that FDGlcU-based imaging could provide a convenient approach for real-time evaluation of the inhibitory efficiency of antibiotics or other medicinal treatments in reducing intestinal bacterial βG activity.

## Results

### *In vitro* hydrolysis of FDGlcU by bacterial βG in BL21 cells

**Figure 1:**
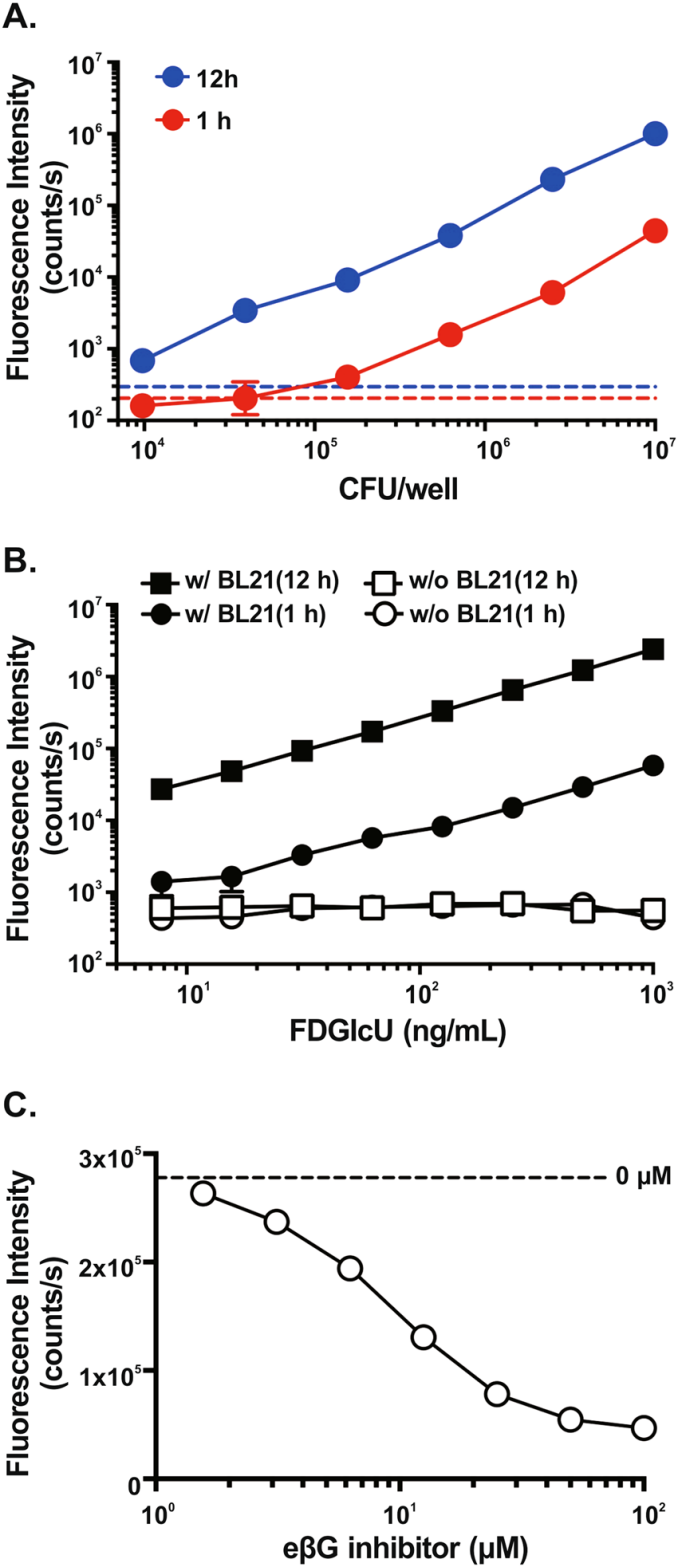
Hydrolysis of FDGlcU in cell-based assays and inhibition by the eβG inhibitor. (**A**) Fluorescence intensity of 0.5 μg/mL FDGlcU hydrolyzed by various amounts (CFU/well) of BL21 cells after 1-hour (red dots) or 12-hour (blue dots) incubations. Dashed lines indicate the absence of BL21 cells in the 1-hour (red line) or the 12-hour (blue line) incubation. (**B**) Fluorescence intensity of various concentrations of FDGlcU hydrolyzed by 10^7^ CFU/well of BL21 cells after 1-hour (●) and 12-hour (■) incubations. (☐) and (○) indicate the absence of BL21 cells. (**C**) Fluorescence intensity of 1 μg/mL of FDGlcU hydrolyzed by 6.25 × 10^5^ CFU/well of BL21 cells in the presence of the eβG inhibitor at various concentrations (μM) after the 12-hour incubation. The dashed line indicates the absence of the eβG inhibitor. Error bars indicate SEM.

The correlation between the fluorescent intensity of FDGlcU and the enzyme activity of bacterial βG was investigated by *in vitro* cell-based assays. Intestinal bacterial βG was represented by *Escherichia coli* (*E. coli*) BL21 strain. Samples of 0.5 μg/mL of FDGlcU were incubated with different amounts of BL21 cells. The fluorescent intensity of hydrolyzed FDGlcU in 1-hour and 12-hour incubations is shown in Fig. 1A. A significant increase in the signal was detected in 6.25 × 10^5^ colony-forming units (CFU)/well of BL21 cells compared with the FDGlcU alone group (p = 0.0015) in the 1-hour incubation. At the 12-hour time point, the fluorescent signal and the number of BL21 cells showed a linear correlation in the log-log plot (r^2^ = 0.9937), and a significant increase of signal was detected in 9.77 × 10^3^ CFU/well of BL21 cells compared with the FDGlcU-alone group (p < 0.0001), indicating that, in the presence of bacterial βG, FDGlcU can be continuously activated for 12 hours. To investigate the minimum concentration of FDGlcU required to produce a detectable fluorescent signal, variant concentrations of FDGlcU were added to 10^7^ CFU/well samples of BL21 cells, followed by 1-hour and 12-hour incubations. The lowest concentration (7.81 ng/mL) of FDGlcU produced a significantly increased signal compared with the BL21 cells-alone group in the 1-hour incubation (p < 0.0003). In the 12-hour incubation, the signal showed a linear correlation with the FDGlcU concentration (r^2^ = 0.9996) (Fig. 1B). In addition, FDGlcU can be kept in a nonfluorescent state during the incubations in the absence of bacterial βG (w/o BL21 (1 h) vs. w/o BL21 (12 h), p = 0.3402) (Fig. 1B). Then, we tested the specificity of the βG probe by pre-treatment BL21 cells with various concentrations of the eβG inhibitor before a 12-hour incubation with FDGlcU (Fig. 1C). The eβG inhibitor displayed a concentration-dependent inhibitory effect on the hydrolysis of FDGlcU by bacterial βG. The relative IC50 of the eβG inhibitor in the cell-based assay was calculated to be 9.24 μM. The concentrations of the eβG inhibitor ranging from 1.6 μM to 100 μM did not affect the growth of BL21 after a 12-hour incubation (p = 0.081) (see Supplementary Fig. S1A) and did not influence the fluorescent signal of FDGlcU (p = 0.1789) and fluorescein (p = 0.3920) (see Supplementary Fig. S1B). These data showed the capability of FDGlcU to quantify the enzymatic activity and inhibition of βG in live bacteria.

### *In vivo* time-lapse imaging of bacterial βG activity

**Figure 2:**
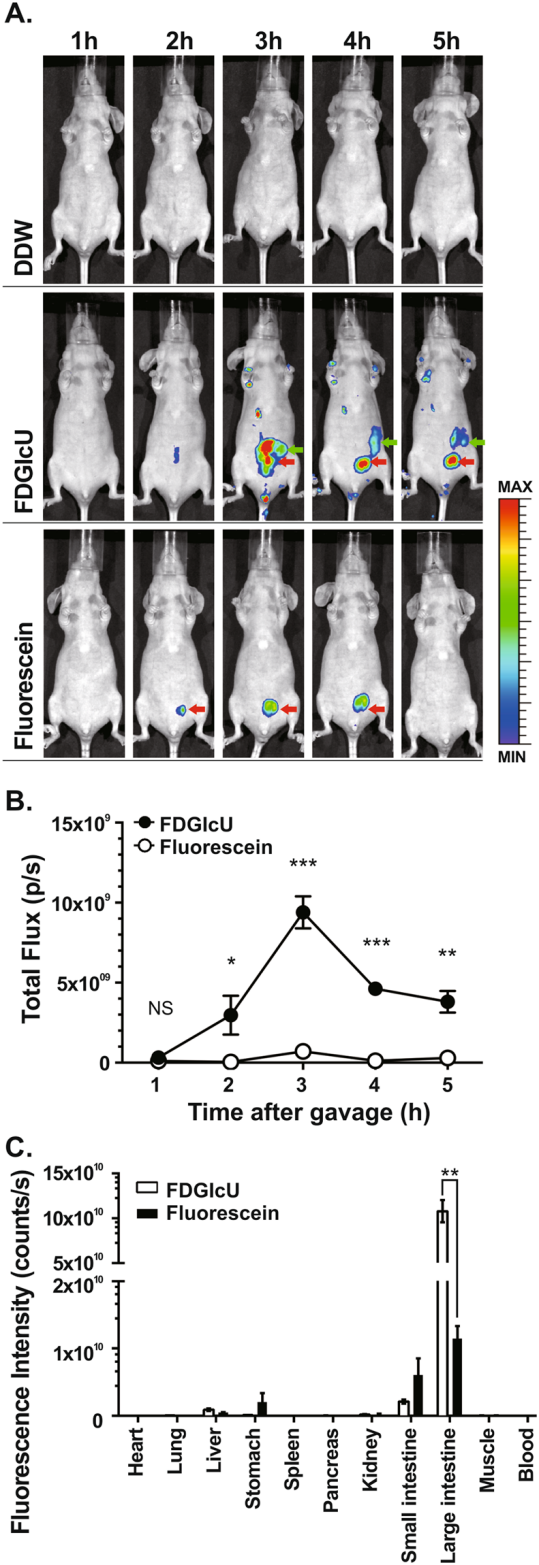
*In vivo* optical imaging of βG activity by FDGlcU. (**A**) Serial whole-body images at indicated time points after oral gavage with DDW (top), FDGlcU (middle), and fluorescein (bottom). Green arrows indicate intestine and red arrows indicate bladder. (**B**) Total flux in abdomens of mice gavaged with FDGlcU (●) (n = 5) or fluorescein (○) (n = 5). (**C**) The regions of interest in different organs and tissues after oral administration of FDGlcU (n = 3) or fluorescein (n = 3) for 3 hours were analyzed with Living Image software. Error bars indicate SEM. NS, no significant difference. *P value < 0.05. **P value < 0.01. ***P value < 0.001.

To test whether bacterial βG can be noninvasively detected in the murine intestine by FDGlcU combined with optical imaging, mice were gavaged with 7.3 μmol/kg of FDGlcU, fluorescein or distilled deionized water (DDW) as a negative control and the whole-body images were sequentially imaged every 1 hour for 5 hours with an IVIS^®^ spectrum optical imaging system. In the whole-body images, a significant fluorescent signal was detected in the abdomens of the mice gavaged with FDGlcU compared with the DDW group mice or the fluorescein group mice, with the signal remaining until the 6-hour time point (Fig. 2A). Fluorescein hydrolyzed from FDGlcU also accumulated in the bladder of the mice gavaged with FDGlcU. In the mice gavaged with fluorescein, the fluorescent signal in the abdomen exhibited no significant change, however bladder exhibited a small signal. The total flux in the abdomens of the FDGlcU-treated mice at the 3-hour time point was the maximum value (9.4 × 10^9^ photons/second (p/s)) and was 13.3-fold higher than that in the fluorescein-treated mice (Fig. 2B). Further images would be acquired at the 3-hour time point. To investigate the biodistribution of FDGlcU and fluorescein in mice at the 3-hour point, the fluorescent signal in excised organs, muscle, and blood were measured by optical imaging (Fig. 2C). The fluorescent signals in the large intestines of FDGlcU-treated mice were 9.4-fold stronger than those in fluorescein-treated mice (p = 0.0015). The signals in other organs, muscle and blood of these two groups exhibited no significant differences. Though slight signals could be detected in the liver, stomach, kidney, and small intestines of the FDGlcU-treated mice, these signals were at least 50-fold weaker than the signals in the large intestines. Collectively, these results indicate that FDGlcU, when hydrolyzed by bacterial βG in the murine large intestine, is non-invasively detectable by optical imaging.

### *In vivo* imaging of the inhibition of bacterial βG activity in murine intestine

**Figure 3:**
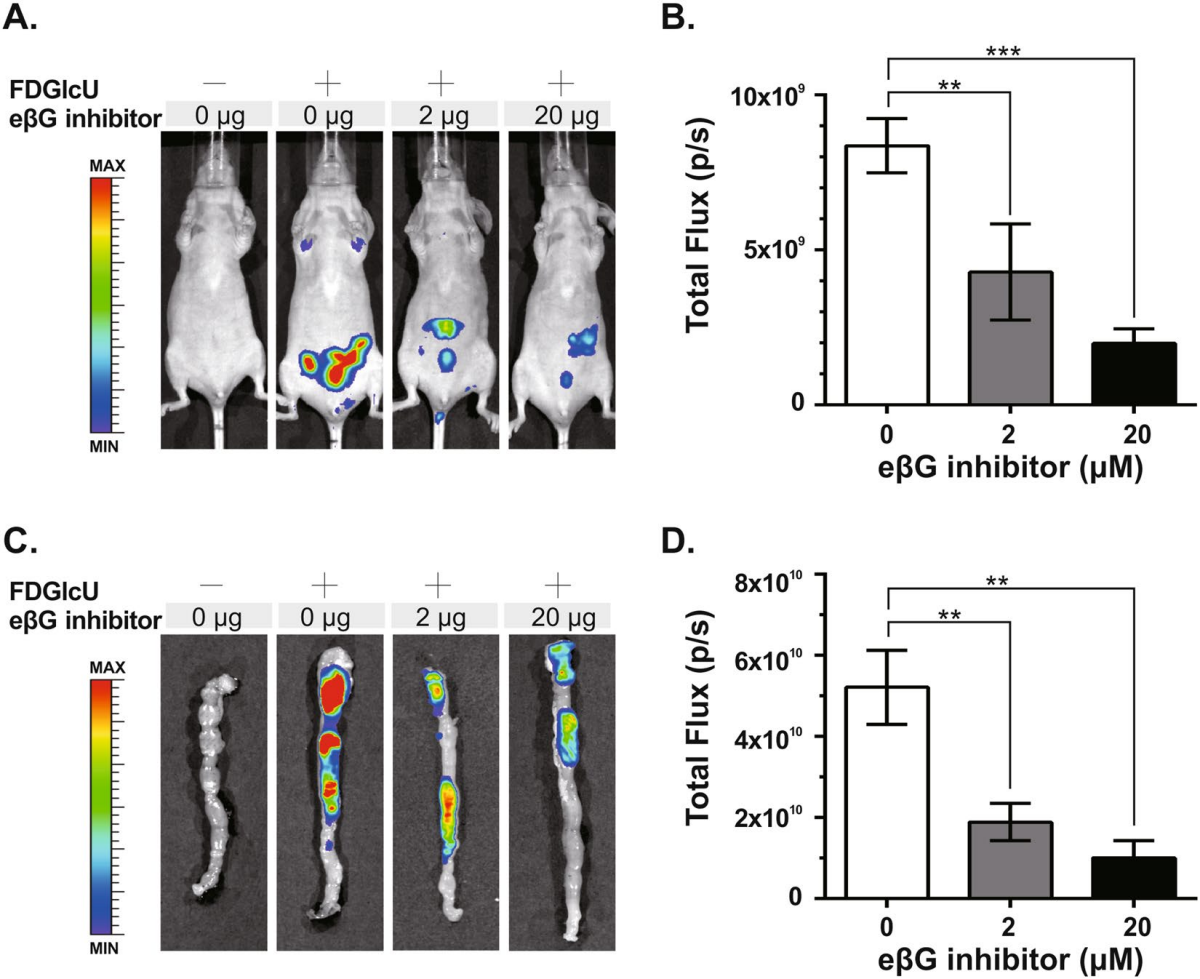
Optical imaging of inhibition of intestinal bacterial βG by the eβG inhibitor. (**A**) Mice were gavaged with the eβG inhibitor (0 μg, 2 μg, or 20 μg) 4 hours before FDGlcU administration, then whole-body imaging was performed 3 hours later. Control mice were gavaged with vehicle solution. (**B**) The fluorescent signal from the murine abdomens of each group (n = 6) was measured. (**C**) Imaging of excised large intestines after whole-body imaging at the 3-hour time point. (**D**) The fluorescent signal from the intestines of each group (n = 6) was measured. Error bars indicate SEM. **P value < 0.01. ***P value < 0.001.

We next applied FDGlcU to noninvasively assess the *in vivo* inhibition ability of the eβG inhibitor on bacterial βG. Mice were orally pre-treated with the eβG inhibitor (0 μg, 2 μg, or 20 μg) 4 hours before FDGlcU administration. Whole-body imaging was performed at the 3-hour point after the FDGlcU administration for maximum signal strength. The fluorescent signal in the abdomens of inhibitor-treated mice was less than that in FDGlcU-alone mice (0 μg of the eβG inhibitor) (Fig. 3A). The low dose (2 μg) and high dose (20 μg) of the eβG inhibitor respectively reduced the fluorescent signal by 2.0-fold and 4.2-fold compared with that produced by FDGlcU alone (p = 0.0015 and p < 0.0001) (Fig. 3B). Large intestines excised from the different treatment groups were also measured by optical imaging (Fig. 3C). The decreases in the fluorescent signals in the excised intestines matched the results for the whole-body images. The total flux in the large intestine excised from the eβG inhibitor (2 μg and 20 μg)-treated mice was decreased by 2.8-fold and 5.2-fold compared with that in the FDGlcU-alone mice (p = 0.0086 and p = 0.0032) (Fig. 3D). Both fluorescent signals in abdomens and excised large intestines were significantly inhibited by the eβG inhibitor in a dose-dependent manner. *In vivo* hydrolysis of the probe revealed the specific inhibition of intestinal bacterial βG activity, which suggested that the selective imaging of bacterial βG activity with FDGlcU could assist in evaluating the optimal doses of inhibitors.

### Real-time imaging of the reduction of intestinal βG activity after an antibiotic treatment

**Figure 4:**
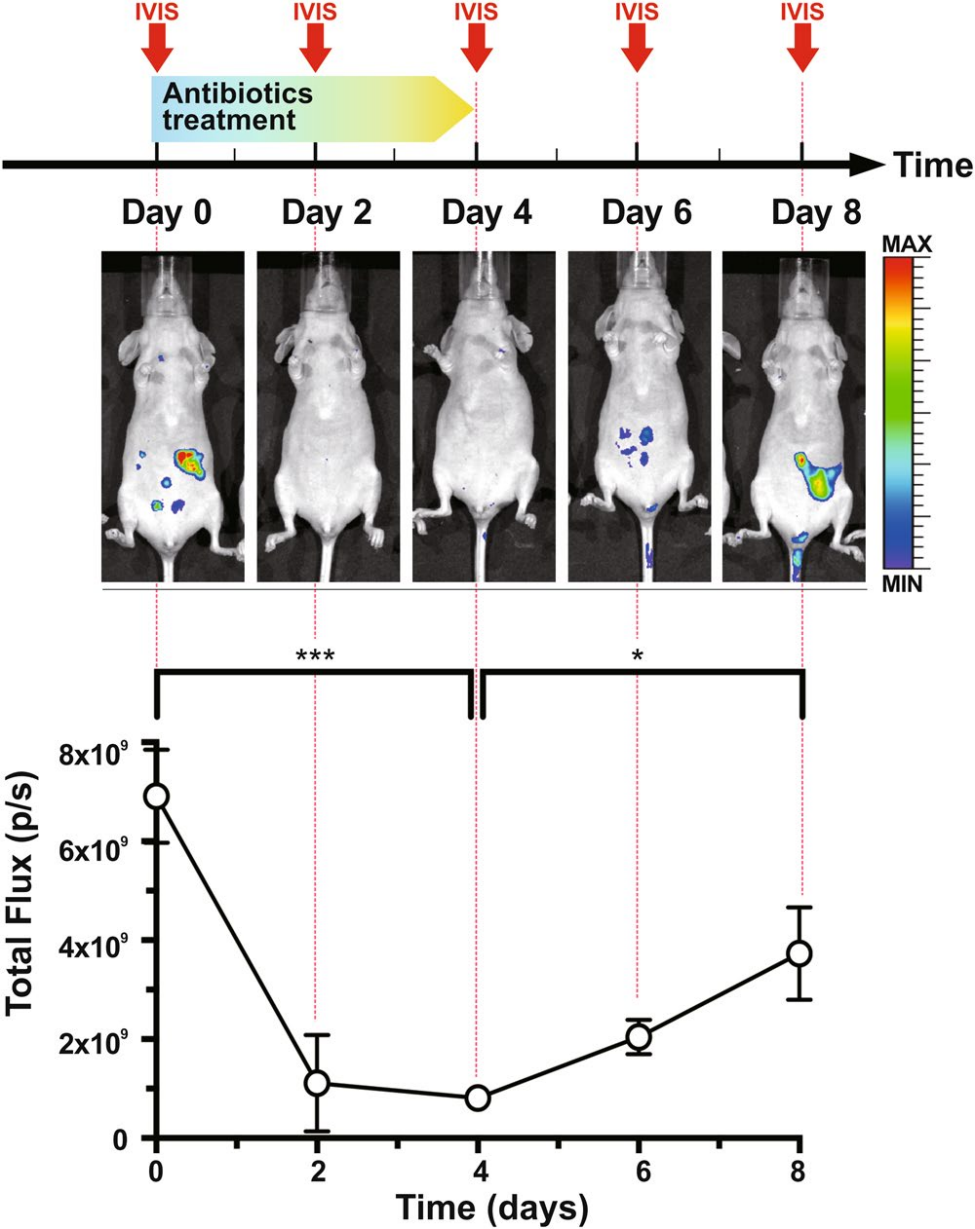
Reduction of intestinal bacterial βG by an antibiotic treatment. Upper panel: Experimental schedule for a 4-day antibiotic treatment and whole-body imaging by the IVIS^®^ imaging system (arrow). Whole-body imaging was performed every 2 days. Antibiotics were orally administered twice daily for 4 days. Middle panel: Imaging of mice at day 0, 2, 4, 6, and 8. Lower panel: Total flux in abdomens of mice (n = 5). Error bars indicate SEM. *P value < 0.05. ***P value < 0.001.

Finally, we used FDGlcU to provide real-time evaluations of the reduction of βG-expressing bacteria and the duration of the inhibitory effects of antibiotic treatment. Mice were gavaged with antibiotics for 4 days and were imaged every 2 days by oral administration of FDGlcU combined with optical imaging (the upper panel of Fig. 4). After antibiotic treatment, the fluorescent intensity in the abdomen dropped and then recovered gradually after the treatment was suspended (the middle panel of Fig. 4). As shown in the lower panel of Fig. 4, the average total flux in the abdomen was 6.9 × 10^9^ p/s on day 0 and then decreased by 8.5-fold to 8.1 × 10^8^ p/s on day 4 (p = 0.0002). On day 8, the total flux was 4.6-fold higher than on day 4 (p = 0.0154). These data suggested that FDGlcU-based imaging offers an effective form of assistance in designing a dosage regimen for antibiotics pretreatment, and that such imaging might be able to estimate the residual enzyme activity.

## Discussion

The results of this study demonstrate the feasibility of applying FDGlcU-based *in vivo* imaging in detecting the enzyme activity of intestinal bacterial βG in a mouse model. The specific hydrolysis of FDGlcU by bacterial βG generated a fluorescent signal whose intensity corresponded to the number of bacteria. For *in vivo* imaging, the most significant fluorescent signal was detected in the intestine as of 3 hours after the administration of FDGlcU. More importantly, the inhibition of intestinal bacterial βG by a specific inhibitor or the reduction of βG-expressing bacteria by an antibiotic treatment was revealed with a non-invasive optical imaging system on consecutive days. Those results suggested that FDGlcU-based imaging could be used to facilitate the real-time measurement of βG activity *in vivo* and to promote the development of preventive therapies for bacterial βG-mediated intestinal damage.

Cell-based assays, which measure βG activity in live bacteria cells, are required for the *in vitro* evaluation of bacterial βG inhibitors. In spectrophotometric assays, PNPG and PHTG are common chromogenic substrates used to develop color after hydrolysis. However, an incubation period of several hours is needed^12^, and the signals for high enzymatic activity may be saturated and be limited to a maximum optical density of 4. Compared with spectrophotometric assays, fluorometric assays have the advantages of greater sensitivity and a wider linear response range (greater than 4 decades). Fluorogenic substrates such as 4-MUG are used in cell-based assays of bacterial βG inhibitors^15^. However, 4-MUG is unsuitable for *in vivo* imaging due to the requirement of a UV excitation source and its short emission wavelength (λ_ex_ = 365 nm/λ_em_ = 455 nm). In contrast, the higher excitation and emission wavelength of FDGlcU (λ_ex_ = 480 nm/λ_em_ = 514 nm) make it suitable for both *in vitro* and *in vivo* assays of βG activity. FDGlcU provides a large detection range (fluorescent intensity: 10^2^–10^6^ counts/s) and high sensitivity for the measurement of βG activity in live bacteria cells. The cell-based assay showed that a fluorescent signal was detectable at 1 hour, and the sensitivity increased with time. Moreover, most optical imaging instruments provide appropriate filter sets for FDGlcU (a GFP filter set was used in this study). Overall, the results of this study demonstrated the utility of FDGlcU as a probe for both *in vitro* and *in vivo* measurements of βG activity.

The detection of βG activity in small animals by non-invasive imaging has been shown in optical imaging, magnetic resonance imaging (MRI), and positron emission tomography (PET). Glucuronide-conjugated probes for MRI and PET were developed to investigate the enzyme activity in neuroinflammation^16^ and tumors^17–20^. Among these imaging systems, optical imaging may be more suitable for the development of bacterial βG inhibitors due to its capacity for rapid acquisition and the imaging of multiple animals simultaneously. FDGlcU has achieved encouraging results in the optical imaging of exogenous βG reporter genes expressed by xenogeneic tumors^21^, an oncolytic virus^22^, and tumor-targeting *E. coli* ^15^ in subcutaneous tissue. Thus, we believed that FDGlcU could serve as a potential *in vivo* optical imaging probe for endogenous bacterial βG. This study constitutes the first time that FDGlcU has been applied in imaging the intestinal bacterial βG activity in a mouse model via oral gavage. The wavelength fluorescence (514 nm) produced by the hydrolysis of FDGlcU is strong enough to overcome absorption in small animal tissue. To obtain higher sensitivity in bigger animals such as rats and rabbits, a near-infrared glucuronide probe might be an option for deeper penetration^23^.

By means of FDGlcU, we directly monitored the *in vivo* activity of intestinal bacterial βG. In previous animal studies of bacterial βG inhibitors, the enzyme activity was evaluated by measuring *ex vivo* fecal βG activity^13, 14^. In this approach, labor-intensive procedures such as the collection and drying of feces are required. Also, the enzyme activity in feces is different from that in intestinal content^24, 25^. Moreover, the evaluation of enzyme activity from excised intestines cannot provide continuous data. By comparison, optical imaging can offer the advantages of speed, simplicity, and non-invasive detection. The data for the present study showed that the enzyme activity in the mouse abdomen could be detected as of 3 hours after FDGlcU administration. The biodistribution of FDGlcU in mice at the 3-hour time point shows that activated FDGlcU was mostly exhibited in the feces or the content of the large intestine where βG-expressing bacteria such as *E. coli* abound. In contrast, very low levels of activated FDGlcU were exhibited in other organs and tissues. This may be because the two hydrophilic glucuronides on FDGlcU prevent the penetration of the probe into mammalian cells^26^. In addition, high expression levels of glucuronide transporters on bacterial surfaces allow FDGlcU to easily enter to intestinal bacteria^27^. Also, serial imaging could be performed every two days because of the rapid clearance of fluorescein from living bodies. The measurement of intestinal bacterial βG would thus not require scarification and the excising of intestines. As such, we believe that the FDGlcU-based imaging could be used to facilitate the screening of the *in vivo* inhibition of βG and, in turn, to hasten the development of preventive therapies.

FDGlcU-based imaging is a feasible method by which to efficiently evaluate the *in vivo* effects of various treatments aimed at reducing intestinal bacterial βG activity. In previous studies utilizing the disease model of CPT-11-induced diarrhea, the inhibitory effect against βG was evaluated by the incidence and severity of delayed diarrhea and bloody stool, an approach which is time-consuming; diarrhea often occurred 6–10 days after CPT-11 administration^28, 29^. Also, the observation of diarrhea can only inform researchers of the final therapeutic effect, not the level and duration of enzyme inhibition. In contrast, in this study, FDGlcU-based imaging allowed the enzyme inhibition to be observed as of 7 hours after the administration of the eβG inhibitor, making this approach much faster than the traditional one of diarrhea observation. Because pharmacokinetic and pharmacodynamic properties are crucial for determining effective dosing regimens, the real-time measurement of *in vivo* enzyme activity would be of significant benefit in animal studies of bacterial βG inhibitors. Additionally, FDGlcU-based imaging provides an enzyme activity assay platform for treatments which lack *in vitro* data. Assaying the inhibitory activity of these treatments may require an *in vivo* environment or the involvement of metabolic enzymes. For example, previous studies of a probiotic treatment showed that *Lactobacillus casei* strain Shirota improved CPT-11-induced diarrhea^30, 31^, but the *in vivo* inhibitory efficiency of the treatment against βG was unclear. Similarly, loxapine is an antipsychotic drug that showed no inhibitory effect in an *in vitro* βG assay, but its metabolite is believed to have exhibited bacterial βG inhibitory activity in an animal study^32, 33^. Our study also demonstrates that the depletion of intestinal bacteria by antibiotics reduced βG activity. Antibiotics have previously been applied for the prevention of CPT-11-induced diarrhea, but the dosages of antibiotics should be carefully considered to avoid imbalances of microflora and *Clostridium difficile* infections. The minimum dose required for a given antibiotic to reduce βG-expressing bacteria could be determined by FDGlcU-based imaging in animal studies.

In summary, we showed that the *in vivo* enzyme activity of intestinal bacterial βG can be evaluated via optical imaging with FDGlcU in mouse models. In the *in vitro* assay of bacterial βG activity, FDGlcU provides a large detection range and high sensitivity. In mouse models, the optimal timing for *in vivo* imaging is 3 hours after gavage with FDGlcU. Furthermore, FDGlcU-based imaging reveals the reduced activity of bacterial βG affected by a specific inhibitor or antibiotics. We thus believe that FDGlcU-based imaging constitutes a convenient *in vivo* screening platform, one which would speed up the development of novel methods to prevent bacterial βG-mediated intestinal damage during treatment with CPT-11 or other drugs.

## Materials and Methods

### Reagents

FDGlcU was purchased from Thermo Fisher Scientific (Waltham, MA, U.S.A.) and reconstituted in DDW. The eβG inhibitor was purchased from Merck (Cat# 347423) (Darmstadt, Germany), reconstituted in DMSO at 4.255 mg/mL, and then further diluted with DMSO for *in vitro* assay or diluted with DDW to 0.2 mg/mL for *in vivo* imaging. Isoflurane was purchased from Aesica (Queenborough, U.K.). Fluorescein was purchased from Sigma-Aldrich (St. Louis, MO, U.S.A.), reconstituted in DMSO, and then diluted with DDW before use. Ampicillin, neomycin, and vancomycin were purchased from Gold Biotechnology (St. Louis, MO, U.S.A.).

### *In vitro* catalytic hydrolysis of FDGlcU by bacterial βG

Cell-based assays with *E. coli* strain BL21 were used to measure the activity of bacterial βG. In all *in vitro* cell-based assays, phosphate-buffered saline (PBS) (pH 7.5) containing 0.05% (w/v) bovine serum albumin and 1% (v/v) DMSO was used as the sample dilution buffer. BL21 cells collected at an exponential phase (O.D. 600 = 0.5) were washed with cold PBS (pH 7.5). BL21 cells at various cell concentrations (50 μL/well) were incubated with 50 µL of 1 µg/mL FDGlcU at 37 °C for 1-hour and 12-hour incubations in a 96-well plate. To assess the minimum concentration of FDGlcU for production of a detectable fluorescent signal, FDGlcU at various concentrations (50 μL/well) were incubated with BL21 cells (10^7^ CFU/50 µL/well) at 37 °C for 1-hour and 12-hour incubations. To investigate the specific hydrolysis by bacterial βG, BL21 cells (10^7^ CFU/49 µL/well) was pre-treated with the serially diluted eβG inhibitor (1 μL/well) at 37 °C for 30 min, then sequentially incubated with FDGlcU (2 μg/50 μL/ well) at 37 °C for 1-hour and 12-hour incubations. The fluorescent signal in each well was measured using a VICTOR X3 Multilabel Plate Reader (PerkinElmer, Waltham, MA, U.S.A.) with an FITC filter set (λ_ex_ = 485 nm/λ_em_ = 535). Signal values were subtracted by the mean background of the same concentration of BL 21 cells and DMSO without adding FDGlcU.

### Mice

Seven-week-old Balb/c nude mice were purchased from the National Laboratory Animal Center, Taipei, Taiwan. The mice were fed with non-fluorescent diet (Ivid#1 diet) (Oriental Yeast Co., Tokyo, Japan) 2 weeks prior to *in vivo* imaging. All animal experiments were approved by the Institutional Animal Care and Use Committee (IACUC) at Taipei Medical University, and all methods were performed in accordance with relevant governmental and institutional guidelines and regulations.

### Whole-body imaging for intestinal βG activity

On the day of imaging, mice were gavaged with 50 μL of DDW, FDGlcU (7.3 μmol/kg), and fluorescein (7.3 μmol/kg). At indicated time points, the mice were anesthetized with isoflurane and whole-body optical images were acquired using an IVIS^®^ spectrum optical imaging system 200 (PerkinElmer) with a GFP filter set. In a biodistribution study, mice were sacrificed 3 hour after gavaged with FDGlcU or fluorescein. Organs or tissues (heart, liver, spleen, lung, kidney, stomach, small intestine, large intestine, pancreas, and thigh muscle) and blood (100 μL) were obtained and measured by the IVIS^®^ imaging system. For the quantification of fluorescent signals, the total flux from regions of interest (ROIs) measured in the DDW group were subtracted from the raw total flux from ROIs of other groups.

### *In vivo* imaging of enzyme activity suppressed by the eβG inhibitor

On the day of imaging, mice fed with non-fluorescent diet were gavaged with 0 μg, 2 μg, or 20 μg of the eβG inhibitor. After 4 hours, whole-body imaging was performed after an administration of FDGlcU to the mice as described above. Control mice were gavaged with vehicle solution (4.7% (v/v) DMSO) and then DDW. The entire large intestines of those mice were excised, washed with PBS briefly, and then imaged using the IVIS^®^ imaging system.

### Elimination of intestinal bacteria with antibiotics

Mice fed with a non-fluorescent diet received daily supplementation of their drinking water with ampicillin 1 g/L, and were gavaged with 100 μL of neomycin 20 mg/mL and vancomycin 10 mg/mL twice per day for a total of 8 oral administrations. Whole-body imaging with FDGlcU administration was performed once every 2 days by the IVIS^®^ imaging system.

### Statistics

Statistical analysis was performed using Prism 6.0 software (GraphPad Software, La Jolla, CA, U.S.A.). Error bars represent the standard error of the mean (SEM). A comparison of the fluorescent signals of FDGlcU in 1-hr and 12-hr incubations without BL21 cells was analyzed with a two-way analysis of variance (ANOVA), and all other *in vitro* cell-based assays were analyzed with one-way ANOVA. The effects of the eβG inhibitor on *in vivo* imaging was analyzed with two-way ANOVA. The biodistribution study of FDGlcU and Fluorescein, and the *in vivo* imaging of antibiotic treatment were analyzed with two-tailed t-tests. Statistical significance was defined as p < 0.05.

## Electronic supplementary material

Supplemental Information
