# Supplementary material for: Real-time imaging of intestinal bacterial β-glucuronidase activity by hydrolysis of a fluorescent probe
Source: Sci Rep. 2017 Jun 9;7:3142. doi: 10.1038/s41598-017-03252-4 (PMC5466677; doi:10.1038/s41598-017-03252-4)
Supplement: Supplementary file 1 — Supplemental Information [file 41598_2017_3252_MOESM1_ESM.pdf]

Real-time imaging of intestinal bacterial β-glucuronidase activity by hydrolysis of a fluorescent probe

Michael Chen[a], Kai-Wen Cheng[b], Yi-Jou Chen[a], Chang-Hung Wang[a], Ta-Chun Cheng[c], Kuo-Chien Chang[d], An-Pei Kao[e], Kuo-Hsiang Chuang[a, f, g, h, *]

[a] Ph.D. program for the Clinical Drug Discovery from Botanical Herbs, Taipei Medical University, Taipei, Taiwan

[b] Institute of Biomedical Sciences, National Sun Yat-Sen University, Kaohsiung, Taiwan

[c] Center for Biomarkers and Biotech Drugs, Kaohsiung Medical University, Kaohsiung, Taiwan

[d] Department of Orthopedics, Tri-service General Hospital, Taipei, Taiwan.

[e] Stemforce Biotechnology Co., Ltd, Chiayi City, Taiwan

[f] Graduate Institute of Pharmacognosy, Taipei Medical University, Taipei, Taiwan

[g] Master Program for Clinical Pharmacogenomics and Pharmacoproteomics, Taipei Medical University, Taipei, Taiwan

[h] The Ph.D. Program of Translational Medicine, Taipei Medical University, Taipei, Taiwan

*Correspondence:

Dr. Kuo-Hsiang Chuang

E-mail: khchuang@tmu.edu.tw

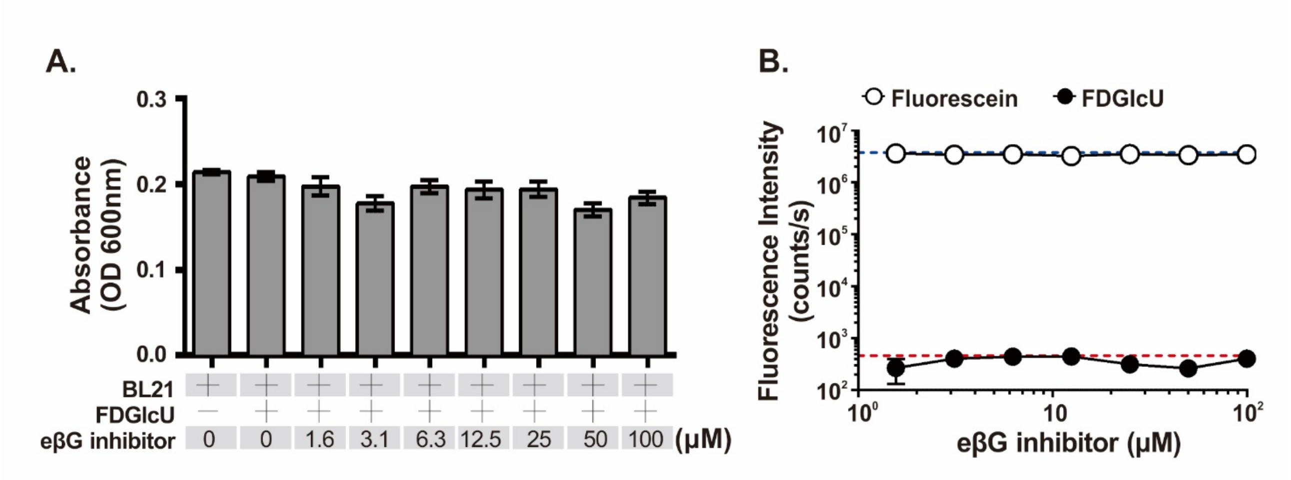

Supplementary Figure S1. The influence of the eβG inhibitor on BL21 cell growth and on fluorescent measurement. (A) The absorbance at 600 nm of BL21 cells after being pre-treated with the serially diluted eβG inhibitor and sequentially incubated with FDGlcU at 37 °C for a 12-hour incubation. (B) The fluorescence intensity of fluorescein (○) and FDGlcU (●) incubated with various concentrations (μM) of the eβG inhibitor but without BL21 cells. The dashed lines indicate the fluorescence intensity of the fluorescein group (blue line) and FDGlcU group (red line) in the absence of the eβG inhibitor. Error bars indicate SEM.

Materials and Methods

The influence of the eβG inhibitor on BL21 cell growth

BL21 cells (10^7 CFU/49 μL/well) were pre-treated with the serially diluted eβG inhibitor (1 μL/well) in a 96-well plate at 37 °C for 30 min, then sequentially incubated with 2 μg/mL of FDGlcU (50 μL/well) at 37 °C for a 12-hour incubation. The amounts of these cells were measured by recording the absorbance at 600 nm.

The influence of the eβG inhibitor on fluorescent measurement

FDGlcU (1 μg/mL; 1.461 μM) or fluorescein (1.461 μM) was incubated with various concentrations (μM) of the eβG inhibitor in the absences of BL21 cells in a 96-well plate. The fluorescent signal in each well was measured using a VICTOR X3 Multilabel Plate Reader (PerkinElmer, Waltham, MA, U.S.A.) with an FITC filter set (λ_{ex} = 485 nm/λ_{em} = 535). Signal values were subtracted by the mean background of phosphate-buffered saline (PBS) (pH 7.5) containing 0.05% (w/v) bovine serum albumin and 1% (v/v) DMSO.
